# Supplementary material for: Metal-Cation-Induced Tiny Ripple on Graphene
Source: Nanomaterials (Basel). 2024 Oct 2;14(19):1593. doi: 10.3390/nano14191593 (PMC11477897; doi:10.3390/nano14191593)
Supplement: Supplementary file 1 [file nanomaterials-14-01593-s001.zip › nanomaterials-3183551-supplementary.pdf]

Supplementary Information for

Metal cation-induced tiny ripple on graphene

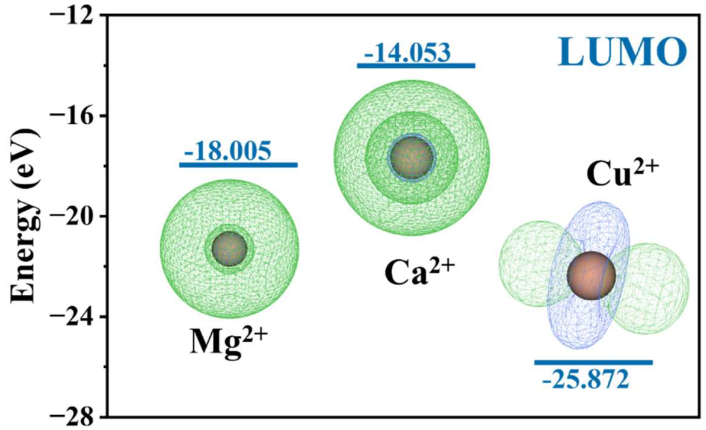

Figure S1. The lowest unoccupied molecular orbital (LUMO) energies of Mg^{2+}, Ca^{2+}, and Cu^{2+} ions, indicated by cyan horizontal lines. The insets display the spatial charge distributions of the LUMO orbitals at an isosurface value of 0.5.

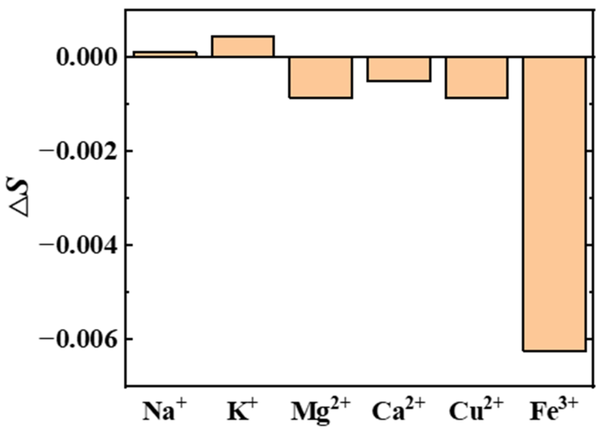

Figure S2. In-plane deformation ($\triangle S$) of the ripple in graphene induced by various metal cations.

The values of $\triangle S$ for graphene with the adsorption of Na^+, K^+, Mg^{2+}, Ca^{2+}, Cu^{2+} and Fe^{3+} are 0.01%, 0.04%, -0.09%, -0.05%, -0.09% and -0.62%, respectively (Figure S1). All values are less than 1%.

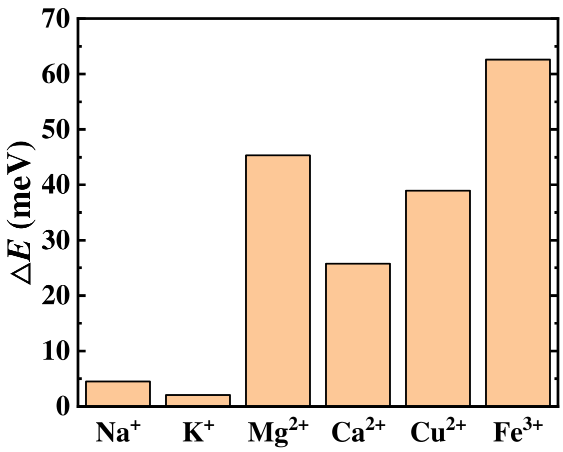

Figure S3. Energy difference ($\triangle E$) between rippled graphene with the adsorption of various metal cations and flat graphene without cation adsorption.

The energy differences ($\triangle E$) between the rippled graphene with the adsorption of Na^{+}, K^{+}, Mg^{2+}, Ca^{2+}, Cu^{2+}, and Fe^{3+} and the flat graphene without cation adsorption are 4.49, 2.03, 45.29, 25.76, 36.19 and 62.64 meV, respectively.

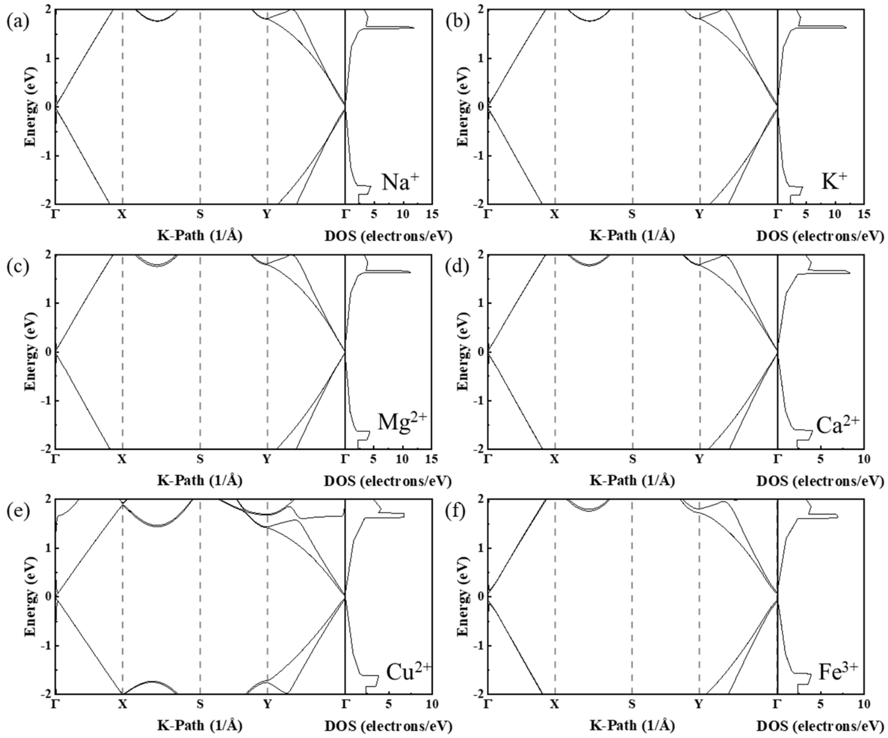

Figure S4. (a)-(f) Band structures and density of states (DOS) for rippled graphene sheets induced by Na^{+}, K^{+}, Mg^{2+}, Ca^{2+}, Cu^{2+}, and Fe^{3+}, respectively. E_g represents the bandgap.

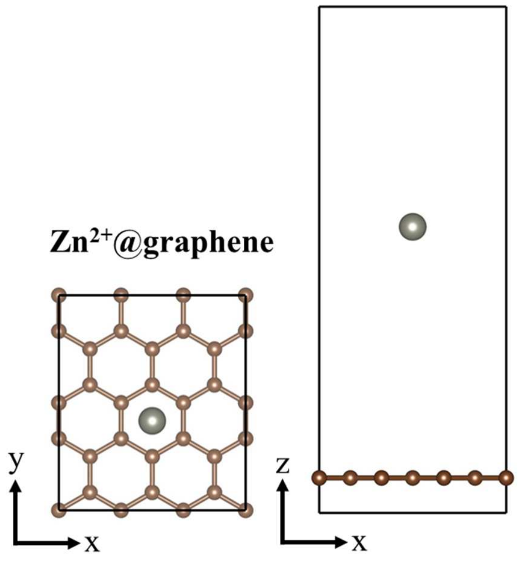

Figure S5. Structure configuration of Zn^{2+} adsorbed on a graphene sheet.

As shown in Figure S4, the vertical distance of Zn^{2+} to graphene sheet is 9.92 Å, which is far larger than that of other cations (less than 3 Å). The ion adsorption energy (E_i) for the system with adsorption of Zn^{2+} is -316.2 kcal/mol, which is comparable to that of Cu^{2+} and about two times of that for Mg^{2+} and Ca^{2+}. The numbers of transferred electrons from the graphene sheet to the unoccupied valence orbits of Zn^{2+} is 1.174 e, which is comparable to that of Cu^{2+}, and about three times of that for Mg^{2+} and Ca^{2+}. The value of deformation in the z direction ($\triangle Z$) for rippled graphene sheet induced by the adsorption of Zn^{2+} is 0.004 Å, which is far less than that of Mg^{2+}, Ca^{2+}, Cu^{2+}, while the relative area change of the rippled graphene compared to flat graphene ($\triangle S$) is 0.61%. Therefore, the ripple of graphene induced by Zn^{2+} is far smaller than that of other divalent cations such as Mg^{2+}, Ca^{2+}, Cu^{2+}.
